# Radiomic analysis of cohort-specific diagnostic errors in reading dense mammograms using artificial intelligence

**DOI:** 10.1093/bjr/tqae195

**Published:** 2024-10-09

**Authors:** Xuetong Tao, Ziba Gandomkar, Tong Li, Patrick C Brennan, Warren M Reed

**Affiliations:** Discipline of Medical Imaging Science, Faculty of Health Sciences, Western Ave, Camperdown NSW 2050, Australia; Discipline of Medical Imaging Science, Faculty of Health Sciences, Western Ave, Camperdown NSW 2050, Australia; The Daffodil Centre, The University of Sydney, A Joint Venture with Cancer Council NSW, Sydney, NSW 2006, Australia; Sydney School of Public Health, Faculty of Medicine and Health, The University of Sydney, Sydney, NSW 2006, Australia; Discipline of Medical Imaging Science, Faculty of Health Sciences, Western Ave, Camperdown NSW 2050, Australia; Discipline of Medical Imaging Science, Faculty of Health Sciences, Western Ave, Camperdown NSW 2050, Australia

**Keywords:** artificial intelligence, diagnostic errors, diagnostic performance, dense mammography, mammography

## Abstract

**Objectives:**

This study aims to investigate radiologists’ interpretation errors when reading dense screening mammograms using a radiomics-based artificial intelligence approach.

**Methods:**

Thirty-six radiologists from China and Australia read 60 dense mammograms. For each cohort, we identified normal areas that looked suspicious of cancer and the malignant areas containing cancers. Then radiomic features were extracted from these identified areas and random forest models were trained to recognize the areas that were most frequently linked to diagnostic errors within each cohort. The performance of the model and discriminatory power of significant radiomic features were assessed.

**Results:**

We found that in the Chinese cohort, the AUC values for predicting false positives were 0.864 (CC) and 0.829 (MLO), while in the Australian cohort, they were 0.652 (CC) and 0.747 (MLO). For false negatives, the AUC values in the Chinese cohort were 0.677 (CC) and 0.673 (MLO), and in the Australian cohort, they were 0.600 (CC) and 0.505 (MLO). In both cohorts, regions with higher Gabor and maximum response filter outputs were more prone to false positives, while areas with significant intensity changes and coarse textures were more likely to yield false negatives.

**Conclusions:**

This cohort-based pipeline proves effective in identifying common errors for specific reader cohorts based on image-derived radiomic features.

**Advances in knowledge:**

This study demonstrates that radiomics-based AI can effectively identify and predict radiologists' interpretation errors in dense mammograms, with distinct radiomic features linked to false positives and false negatives in Chinese and Australian cohorts.

## Introduction

Screening mammography acts as the front-line in breast cancer detection and early diagnosis. However, its actual benefits are highly dependent on diagnostic accuracy, where the predominant element is the observer performance in mammogram reading. With accurate interpretation, screening can reduce breast cancer mortality rate by 30%-40%.^1^ However, reading mammograms is challenging with diagnostic errors often reported. A cohort study revealed that the overall chance of experiencing one or more false positives exceeded 50% following a decade of yearly breast screenings, and 7%-9% of these women screened received false positive biopsy suggestions.^2^ Diagnostic errors are more frequently reported in dense breasts as their mammograms tend to be more complex and challenging to interpret compared to non-dense breasts.^3^^,^^4^

Many attempts have been made to minimize diagnostic errors, such as optimizing the double reading strategy that proposes to pair readers with complementary visual search patterns instead of arbitrary allocation, and developing computer-aided breast cancer detection tools that can provide readers with second opinions on cancer detection.^5^^,^^6^ However, it is not straightforward to implement paired double reading in practice since the visual search data of individuals is rarely available, and the individual search behaviours change over time as they gain more diagnostic skills.^5^ For computer-aided cancer detection tools, the results are often associated with high false positive errors, and even though more recent machine learning innovations around diagnosis have still been evaluated for efficacy, they are currently considered at best a supplement rather than a replacement for human.^7^ Additionally, the above methods cannot directly improve reader’s interpretive ability, which is the fundamental element in contemporary practice performance.

To enhance observer performance, the first step is to identify any reading weaknesses, specifically focusing on mammographic appearances that pose particularly challenges for correct detection. By recognizing these characteristics, tailored training materials can be developed and provided. This can be achieved by retrospective analysis of reader’s previous reading data using artificial intelligence (AI), which can map image appearances against diagnostic errors.^8–10^ The image appearances are quantified before being fed to AI models, and a popular approach is radiomics. Radiomics involves the high-throughput extraction of image features, converting them into quantitative data that can be mined. Radiomics are related to observer performance.^11–13^ For example, a study proposed a model that maps the mammographic appearances of breast masses to the likelihood of false negative errors, and these masses were represented by computer-extracted radiomic features.^8^ This personalized model helps identify masses that are particularly difficult to detect successfully at the individual level. However, putting such models into practice is still challenging, because individual reading data is rarely available and often insufficient to develop accurate AI models which require a large amount of data.^14^

To overcome this limitation, we developed customized models capable of analysing the common errors for a geographic cohort of readers, which can be applied to other readers sharing similar geographic characteristics without the need for their reading data to be available. Increasing evidence suggests that developing cohort-based AI models to predict specific local areas on mammograms where readers in a particular cohort may make incorrect assessments is feasible.^15^ Studies have shown that diagnostic errors are not random but fall into a pattern for readers with similar characteristics, such as population demographics and levels of expertise.^16–19^ For example, radiologists practicing in Vietnam are more likely to miss lesions with spiculated stellate appearances, while Australian readers are more likely to miss lesions with non-specific density.^16^^,^^17^ Also, radiologists with less experience have more difficulty in detecting smaller lesions successfully than those with more experience, especially lesions with indefinite margins.^18^^,^^19^

The study aimed to investigate the presence of similarities in interpretation errors among radiologists with comparable geographic characteristics when interpreting dense screening mammograms. Additionally, the study aimed to determine whether an AI model could effectively capture these shared errors using radiomics. There were two objectives: (a) analysing the performance of radiologists with the same geographic population characteristics in identifying normalities and detecting malignancies when reading dense screening mammograms; (b) proposing a machine learning framework to recognize the local mammographic areas that are particularly challenging to diagnose accurately for the specific radiologist cohort.

## Methods

### Ethics approval

The retrospective study was ethically approved by the Human Research Ethics Committee of XX (Approval Number: XX) and adheres to all relevant guidelines and regulations. Permission to access the data of Chinese and Australian radiologists was granted from the Breast Screen Reader Assessment Strategy (BREAST) committee. Individual consent from each radiologist was waived, as the BREAST had previously obtained informed consent from all participants before they commenced the test set evaluation.

### Acquisition of data sets

We collected a database of readings from the BREAST, an online mammography training platform, including a mammography test set and radiological assessment from two geographically independent reader cohorts.^20^ The test set had 60 dense mammographic cases, oversampled from the screening population. Among these, 40 cases were of normal classification, confirmed by 2-year negative screening reports, while 20 cases were biopsy-proven malignancies (lesions = 21). The two-year follow-up period was sufficient to confirm the absence of cancer, effectively ruling out interval cancers typically detected within two years following a normal screening mammogram.^21^ The choice of 60 mammography cases with a split of 20 positive and 40 negative cases in the BREAST set is carefully calibrated to reflect specific considerations, ensuring a balance in statistical power to detect significant differences or patterns in radiologist performance, and feasibility within the available resources and time constraints of the radiologists.^22^

All mammograms were acquired using digital technology and underwent de-identification. The cases were deliberately chosen to ensure high image quality and to present challenges that would be beneficial for educational and self-assessment purposes.^22^ Each case contained standard craniocaudal (CC) and mediolateral oblique (MLO) projections of both breasts. The location and subtypes of the lesions in cancer cases were confirmed by consensus of at least two senior radiologists, each with over 20 years of expertise in breast imaging, based on current and previous mammography, histopathological reports, and any additional imaging. The radiologist involved in this confirmation did not participate in the subsequent readings. Information on mammographic density categorized by the Breast Imaging Reporting and Data System (BIRADS)^23^ and lesion subtypes is shown in Table 1. According to commonly accepted criteria, BIRADS A and B are generally associated with low density, while BIRADS C and D indicate high density.^23^ As our study specifically focuses on evaluating reader performance in the context of dense breasts, we excluded the two cases from the BIRADS B from our analysis to ensure a more specific investigation into the diagnostic challenges related to dense breast mammograms.

**Table 1.** Details of mammographic cases analysed in this study.

Calcification	Subgroup	Cancer-free cases	Cancer cases
Breast density (*n* = 60)			
	BIRADS A	0/40	0/20
	BIRADS B	0/40	0/20
	BIRADS C	30/40	15/20
	BIRADS D	8/40	5/20
Lesion types (*n* = 21)			
	Stellate		9/21
	Architectural distortion		2/21
	Calcification		2/21
	Discrete mass		5/21
	Non-specific density		3/21

A total of 36 registered radiologists from China (cohort A, *n* = 20) and Australia (cohort B, *n* = 16) reviewed this test set. These independent geographical cohorts were selected to represent populations with varying breast screening participation rates and differences in radiology training. This selection facilitates an understanding of the diagnostic efficiency of dense screening mammography across radiologists from China and Australia, providing a significant comparison and learning opportunity between the two counties.

In China (cohort A), mammography screening is conducted on a mass and opportunistic basis with a low level of participation, whereas Australia (cohort B) runs a country-wide population-based screening program with a high rate of participation. The lower participation in breast screening in China can be attributed to various factors, including inadequate awareness, fatalism, stigma, financial constraints, and limited access to screening and diagnostic facilities.^24^ These cultural and systemic influences have contributed to diagnoses of breast cancer at later stages, often with larger lesions compared to those detected through regular screening.^25^ Consequently, Chinese radiologists may be more accustomed to detecting late-stage, large-sized tumours (greater than 30 mm) compared to their Australian counterparts.^26^

Regarding radiology training, formal training programs are limited to selected metropolitan areas in China.^27^ Compared to China, radiologists in Australia involve a more structured and subspecialty-focused training pathway, which emphazises passing national and specialty examinations after residency training.^28^ The radiologists in each cohort exhibit diverse levels of expertise. The years of experience in reading mammograms span from 1 to 31 years for cohort A, with a median experience of nine years. In cohort B, the range of experience is from 0 to 32 years, with a median of 8.5 years. The weekly workload for mammography cases varied widely, ranging from fewer than 20 cases to over 200 cases. In cohort A, the majority of readers reviewed between 20 and 100 cases per week, whereas more than half of the readers in cohort B reviewed fewer than 20 cases per week.

Each reader independently evaluated all cases in the standard mammography reading environment, either using the online BREAST platform during a workshop/conference or in their regular clinic where they typically worked, potentially in either Australia or China. Mammograms were displayed in DICOM format through the Picture Archiving and Communication (PACS) system on two calibrated medical grade five-megapixel monitors. Additionally, the BREAST software provided identical images in JPEG format for readers to mark and rate all suspicious lesions. Standard image processing operations were provided such as panning and zooming.^22^ The classification followed the Royal Australian and New Zealand College of Radiologists (RANZCR) guideline.^29^ There are variations between the RANZCR and BIRADS systems, but they are generally interchangeable.^30^ Annotations with a RANZCR score of <3 were classified as “negative”, and those with scores of 3 or higher were deemed “positive”. We considered that cancer lesion was marked correctly if the distance between the positive annotation and the lesion centre was no greater than the radius of the lesion, which was around 12.5 mm for the cancers in this test set. Details of the reading process have previously been described elsewhere.^31^

### Diagnostic error analysis

**Figure 1.**
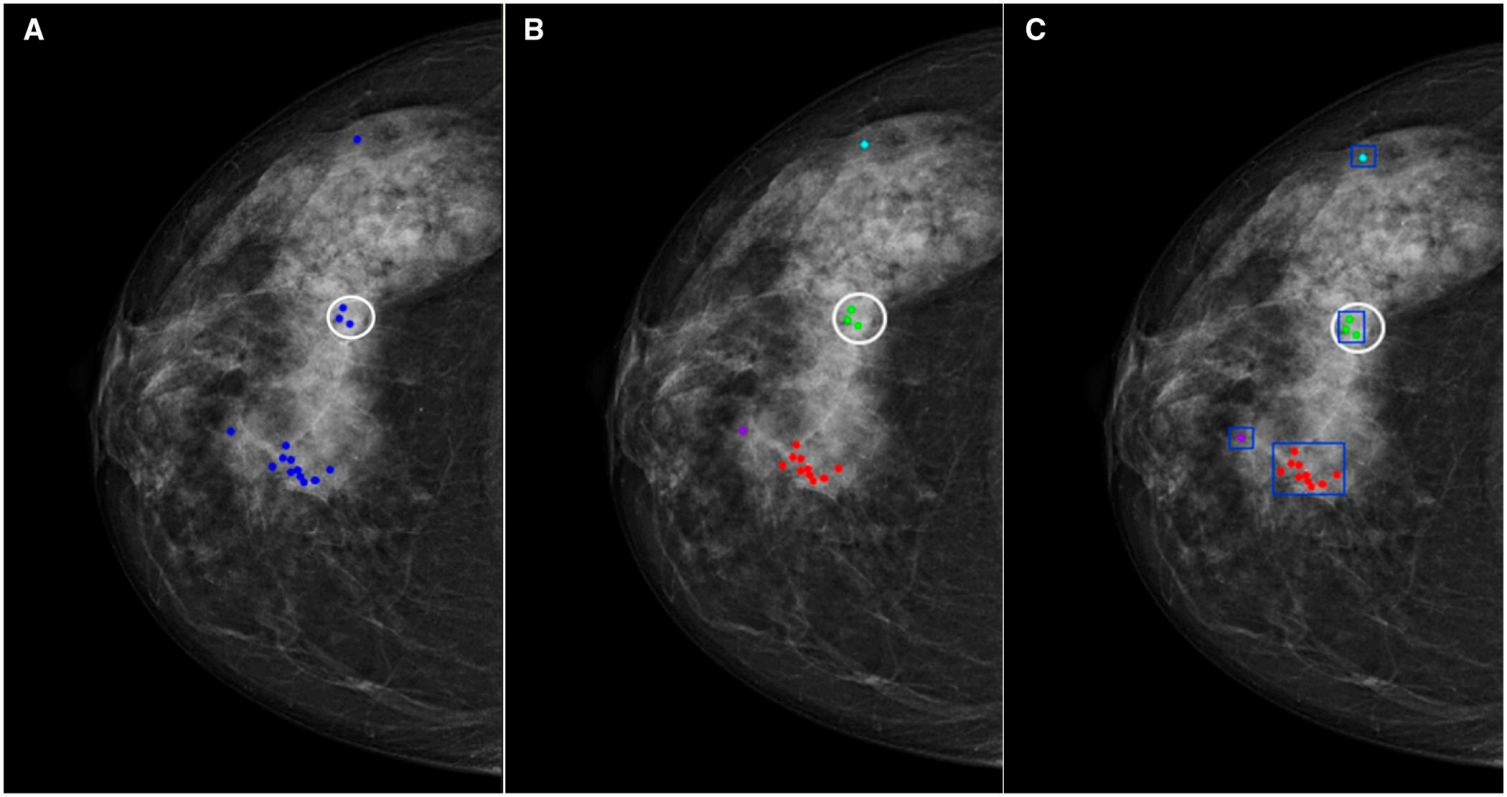
An illustration of a cancer case with all positive markings from one cohort. (A) The blue markings indicate the positive annotations with ratings above 2, and the white circle indicates the cancer border. (B) All positive markings were clustered, and each cluster was given a unique colour. (C) A rectangular box was drawn manually outside each cluster.

All the local areas suspected of cancer were identified, including normal areas reported as cancer and the malignant areas actually containing cancer. To achieve this, we first pooled the positive annotations (ie those rated above 2) from all readers in each cohort and visually displayed them on mammograms. For cancer cases, the malignant lesions were also denoted (Figure 1A). Then, we clustered these positive annotations so that the distance from each point in a cluster was no greater than 250 pixels (Figure 1B).^32^ The threshold was chosen empirically to align with the average diameter of breast lesions found within the two test sets. To ensure accuracy, another researcher, who was blinded to the pathological outcomes, manually adjusted cases where different pixel sizes were encountered. Final clustering decisions were reached by consensus in cases where conflicts arose.

Subsequently, we drew a rectangular box outside each cluster, extending the border of the box 10 pixels beyond the nearest annotation point to ensure all relevant annotations were included (Figure 1C). This box indicated the local areas where at least one of the readers from a specific cohort made a false response. Rather than delineating individual questionable structures or malignant tumours, we opted to use a rectangular box to indicate the suspicious local areas, acknowledging that the characteristics of the surrounding tissues may also influence how radiologists interpret cancer findings. All authors conducted thorough reviews and validated the clustering and delineations to ensure accuracy and appropriateness. This was done using MATLAB R2021a (Mathworks, MA, United States). Each reader might give multiple annotations, and thus the overall marking count may exceed the number of readers within some cases.

To ensure the generality of the cohort-based model, radiologists with the most different reading patterns from the rest of the readers in each cohort were identified and then excluded from model construction using agglomerative hierarchical clustering (HC).^33^ HC can successively group readers with similar responses to each identified local area and generate dendrograms that visually illustrated the hierarchical relationship of radiologists’ reading behaviours.^33^ We performed HC for the normal and the cancer-containing areas, respectively, in each cohort, as models were built to analyse the false positive and false negative errors separately. To achieve this, we flagged the false response as “1” and the correct response as “0” and calculated the Euclidean distance between readers’ responses as a measure of similarity. Specifically, the response to a normal area was considered false positive if the reader left a positive mark and true negative if only a negative mark or no mark was given. Likewise, the response to a cancer-containing area was considered false negative if the reader left a negative or no mark and true positive if a positive mark was left. Then Ward’s minimum variance method was applied as the linkage to merge small groups into big ones that have the minimum variance within each group. From the dendrogram, the reader(s) in the outer branch(s) was/were identified as the outlier(s) with the most dissimilar reading behaviours which was/were excluded from the specific cohort. By doing this, the local areas marked solely by the outlying reader(s) were also removed. This made the final number of readers be 14 and 20 in cohort A in the construction of models analysing false positive and false negative errors, respectively, and be 14 and 16 in cohort B.

Subsequently, the remaining local areas were divided into two cohort-specific diagnostic difficulty categories, “easy” and “difficult”, based on the median value of the number of false responses in each area of interest. Normal and cancer-containing areas were classified according to false positive and false negative responses, respectively. Specifically, normal areas with the number of false positive responses above the median value were classified as “difficult”, and likewise, cancer-containing areas with the number of false negative responses above the median value were classified as “difficult”. Areas with the number of false responses below or equal to the median value were classified within the “easy” group.

### Radiomic pipeline

#### Image segmentation and radiomic feature extraction

Subsequently, we drew a rectangular box outside each cluster, extending the border of the box 10 pixels beyond the nearest annotation point to ensure all relevant annotations were included (Figure 1C). This box indicated the local areas where at least one of the readers from a specific cohort made a false response. Rather than delineating individual questionable structures or malignant tumours, we opted to use a rectangular box to indicate the suspicious local areas, acknowledging that the characteristics of the surrounding tissues may also influence how radiologists interpret cancer findings. All authors conducted thorough reviews and validated the clustering and delineations to ensure accuracy and appropriateness.^34^ The normalization process standardized the intensity values to have zero mean and unit variance, minimizing the impact of variations in image acquisition settings. To validate the normalization process, we checked the histograms of pixel intensities before and after normalization to ensure uniformity. Resampling was performed to ensure all images had a consistent resolution, which is crucial for the consistent and reproducible extraction of radiomic features. All images were resampled to a pixel spacing of length 0.1 mm using bicubic interpolation.^35^ The resampling process was validated by comparing the spatial resolution of resampled images to their original resolution.

Then, we automatically extracted 201 radiomic features per ROI including first-order statistics from histograms, second-order statistics depicting textures, and higher-order statistics from transformed images:

- First-order (*n* = 28)^34^
- Texture (*n* = 139): grey level co-occurrence matrix (GLCM) (*n* = 88),^36^ grey level run length matrix (GLRLM) (*n* = 6),^37^ grey level sharpness measure (GLSM) (*n* = 6),^38^ grey level difference statistics (GLDS) (*n* = 15),^39^ neighbourhood grey tone difference matrix (NGTDM) (*n* = 15),^40^ statistical feature matrix (SFM) (*n* = 8),^41^ and fractional dimension texture analysis (*n* = 1).^42^
- Filter-based higher-order (*n* = 34): Law’s texture energy measures (*n* = 18),^43^ Gabor filter (*n* = 6), maximum response (MR) filter (*n* = 8), and Fourier-transformed (*n* = 2).^44–46^

It is worth noting that the Gabor and MR filters are wavelet filters known for their sensitivity to both frequencies and orientations. Specifically, we focussed on extracting Gabor filter responses within a single scale, using a sinusoidal carrier wavelength of four pixels per cycle (wavelength = 4) and orientations of 0°, 30°, 60°, 90°, 120°, and 150°. The selection of four-pixel wavelength scale and 0°-150° orientations was supported and validated by the empirical findings from our previous studies, which experimented with a filter bank for forecasting diagnostic mammogram interpretation errors.^47–49^ These studies showed that the Gabor filter's responses at a wavelength of 4 pixels provide the most informative insights for error prediction, likely due to their ability to capture subtle irregularities and false positive regions resembling architectural distortions.^47^^,^^48^

For creating the MR filters, we adhered to Varma et al’s suggestions regarding scale and number of orientations.^50^ The MR8 filter bank originated from a shared Root Filter Set (RFS) comprising 38 distinct filters. The RFS included a Gaussian filter and a Laplacian of Gaussian filter, both exhibiting rotational symmetry with a sigma value of 10 pixels. Additionally, it featured an edge and a bar filters at three scales, with each filter oriented at six different angles within each scale (orientations = 0°, 30°, 60°, 90°, 120°, and 150°).

To ensure rotational invariance, the transformation to the MR8 filter bank retained only the highest filter response across all orientations for the two anisotropic filters. This process reduced the initial 38 responses to eight responses. Thus, while the MR8 filter bank comprised 38 filters in total, it yielded only eight filter responses.

The list of extracted features is listed in Table S1. Feature extraction was performed on MATLAB R2021a (Mathworks, MA, United States).

#### Model building

We introduced two machine learning pipelines that streamline and automate data preparation, feature selection, and model building processes. The pipelines analysed the relationship between the diagnostic difficulty level and the radiomic characteristics derived from the normal and malignant areas, respectively. Specifically, each pipeline was developed on the CC and MLO views separately since radiologists normally rely on both views to reach a final decision, and suspicious findings in one view will affect the reader’s responses to the corresponding area in the other view. In this study, we selected the random forest (RF) algorithm as our model due to its well-documented strengths in handling high-dimensional data, its capability to model complex interactions between features, and its robustness against overfitting.^51^ RF is particularly advantageous in medical image analysis due to its ensemble learning approach, which aggregates predictions from multiple decision trees to improve generalization and accuracy.^52^ Additionally, RF provides intrinsic feature importance measures, enabling us to identify the most significant features contributing to the predictive model.^53^

When configuring the RF model, we optimized key hyperparameters to enhance its performance. Specifically, we used *max_features='sqrt'* to determine the number of features to consider at each split, striking a balance between tree diversity and computational efficiency. Additionally, we conducted experiments with different values for *n_estimators*, which denotes the number of trees in the forest. We tested configurations with 100, 250, 500, and 1000 trees using nested cross-validation, as explained subsequently.

Throughout our model building process, we assessed several alternative algorithms, including logistic regression (LR), support vector machines (SVM), and gradient boosting machines (GBM). These alternatives were evaluated based on their AUC values. After comprehensive evaluation, RF emerged as the most suitable choice for our study, demonstrating superior predictive accuracy, while maintaining computational efficiency.

#### Model evaluation

Within each classification pipeline, we applied nested cross-validation (CV) to ensure robust model evaluation and hyperparameter tuning while mitigating the risk of overfitting and providing an unbiased estimate of the generalization ability.^51^ “The approach comprised two loops: one outer loop for performance assessment and one inner loop for hyperparameter optimization”.^51^ By incorporating nested CV, we were able to optimize the model parameters effectively and obtain a reliable estimate of model performance on unseen data. This rigorous validation process was essential for developing a model that could generalize well across different datasets and settings.

**Figure 2.**
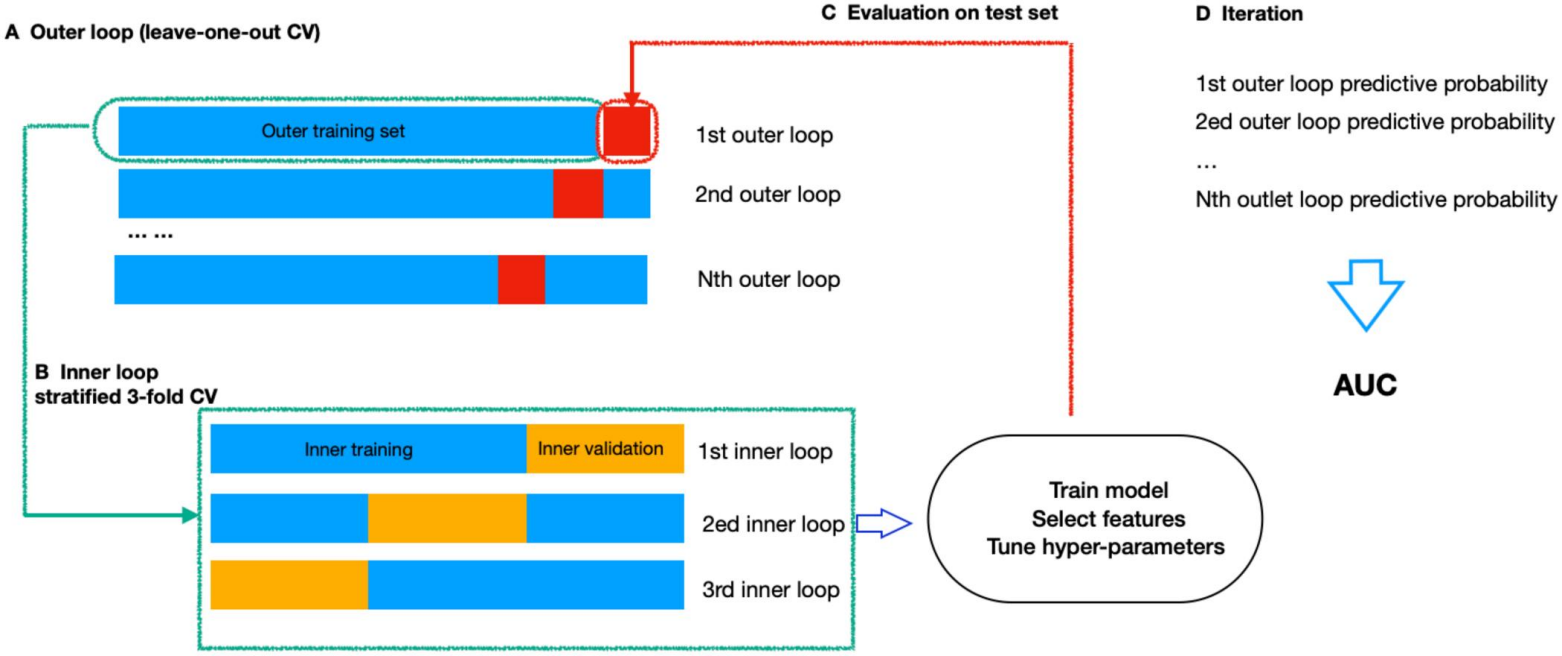
Nested cross-validation illustration. (A) The outer loop contained *k* folds. Of these, (*k* − 1) folds were used as outer training set (blue), while the kth fold served as outer testing set (red). (B) The outer training set was further divided into 3-folds in the inner loop, two formed the inner training set, while one set was left for inner validation. (C) The hyperparameters were optimized in the inner loop and the performance was evaluated in the outer loop. (D) The process outlined in steps A-C was iterated until every sample in the outer loop had been evaluated. Finally, an AUC score, integrating the outcomes of each outer loop, was computed to measure the model performance.

As shown in Figure 2, the outer loop utilized leave-one-out CV, where the dataset was split into *k* folds (where *k* equals the sample size in our study). In each iteration, 1-fold acted as the test set, while the other *k* − 1 folds were used for training. This process was repeated *k* times, ensuring that each data point was used for both training and validation exactly once.

Within each iteration of the outer loop, the inner loop employed repeated 3-fold stratified CV for hyperparameter tuning. The training set from the outer loop was further divided into *m*-fold (where *m* = 3). Each of these *m*-fold served as a validation set, and the rest *m* − 1 folds were used for hyperparameter optimization. This stratified approach ensured that the class distribution was preserved within each fold, providing a balanced representation of the data and improving the reliability of the hyperparameter tuning process.

Specifically, in the inner loop, we utilized synthetic minority oversampling technique (SMOTE) to increase the size of the minority class (ie, the “difficult” class), as the distribution of “easy” and “difficult” classes was imbalanced.^54^ Data imbalance could potentially lead to biased model training, where the classifier might favour the majority class due to its larger representation in the dataset. The use of SMOTE aligned with suggestions in the literature for handling imbalanced datasets in machine learning tasks.^55^

After SMOTE, we standardized the radiomic features and selected features with a variance greater than one. Subsequently, the hyperparameter of RF, specifically the number of trees, was exhaustively searched from 100, 250, 500, to 1000. This inner loop process was repeated three times for each combination of hyperparameters. The hyperparameters that yielded the best average performance across these *m*-fold validations were selected.

The model was trained with the selected hyperparameters on the full training set (from the outer loop) and evaluated on the outer test set. Then performance metrics across all outer loop iterations were aggregated to obtain model’s generalization performance. The performance was measured by the area under the receiver operating characteristic curve (AUC) and accuracy, with an AUC of 0.5 considered as random guessing. To further validate the model, we employed a 2000-time stratified bootstrap to obtain the 95% confidence interval (95% CI) of the AUC.

While nested cross-validation was robust, it had limitations. It was computationally intensive due to multiple rounds of cross-validation. Additionally, random splits may not ensure equal class distribution or feature representation across folds, potentially introducing bias. To mitigate this, we used stratified cross-validation in our study.

The scikit-learn package in Python Version 3.9.4 was employed.

#### Comparison of the discriminative power of features

We also analysed the discriminative power of the radiomic features that contributed the most to the classification tasks. As mentioned above, features with a variance greater than one were selected in each of the inner loops. Next, the remaining features were selected by the RF built-in feature importance method during the repeated nested CV loops. Specifically, within each iteration of the outer loop, features were ranked using the RF model, and the top 10 features were recorded. After completing all iterations, we identified the five features that appeared most frequently across all loops. These features were considered the most discriminative that consistently contributed to the classification implementation. We assessed the stability of feature rankings across repeated nested CV loops to ensure that the features identified as discriminative were consistently influential across different subsets of the data, thereby enhancing the reliability of our feature selection process.

Comparisons were made between the discriminative features from the “easy” and “difficult” categories using the Mann-Whitney *U* test, with two-tailed tests of significance at re employed with significance level of .05. To address multiple comparisons and maintain a lower overall type I error rate, the Bonferroni correction method was applied with a family-wise error rate of .05. This correction is essential in simultaneous multiple testing scenarios to reduce the likelihood of false positive results. Statistical tests were performed using statsModels and SciPy modules in Python version 3.9.4.^56^^,^^57^

## Results

**Figure 3.**
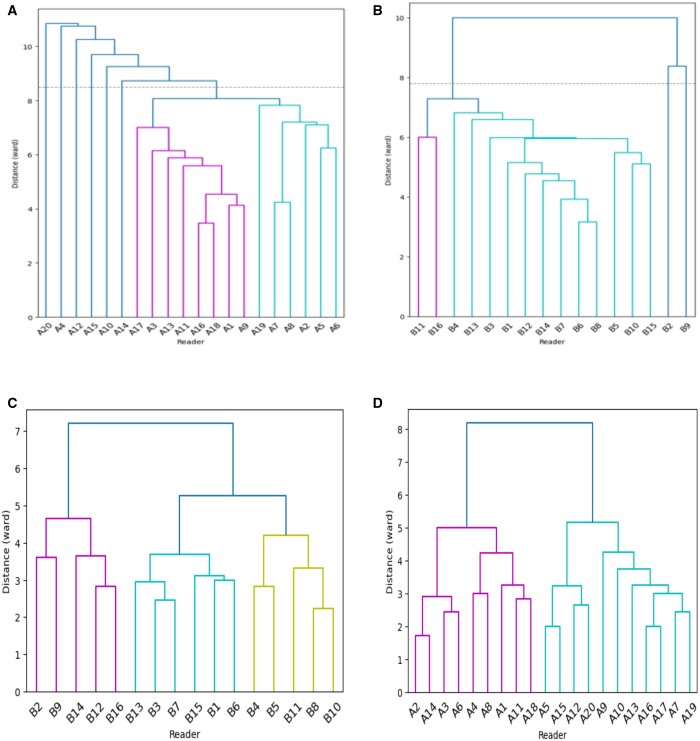
Clustering dendrograms for reading normal and cancer-containing areas for cohort A and B. (A) False positive patterns for cohort A, with six outliers; (B) false positive patterns for cohort B, with two outliers; (C) false negative patterns for cohort A; and (D) false negative patterns for cohort B.

HC analysed the responses of each radiologist to all the investigated local areas suspected of cancer in the 240 images (60 mammographic cases × four views), including the normal areas reported as cancer and the actual cancer-containing areas for each cohort. Figure 3 displays the clustering dendrograms of diagnostic errors for both cohorts. The vertical axis indicated the dissimilarity between groups, and the horizontal axis indicates each individual radiologist. The height at which two clusters merge signifies their similarity, with lower heights indicating higher similarity. Outliers were identified based on the structure of the dendrogram and the clustering distances. Typically, outliers were data points that did not clearly belong to any of the main clusters or were significantly distant from the majority of observations in terms of clustering distances. To ensure consistency and reliability, the interpretation of dendrograms and outlier identification was conducted through consensus among researchers involved in the study.

According to this principle, we found that readers A4, A10, A12, A14, A15, and A20 from cohort A and B2 and B9 from cohort B showed the highest dissimilarity with a different interpretative pattern from the rest of the readers in correctly identifying normal areas. They contributed to more false positive errors than the others. Specifically, readers A4, A10, A12, A14, A15, and A20 reported a total of 82, 77, 87, 55, 73, and 90 normal areas as cancer, while the median false positive responses per reader in cohort A was 41. In cohort B, the median false positive responses per reader was 43, while readers B2 and B9 contributed to a total of 66 and 63 misclassifications. Although readers A4, A10, A12, A14, A15, and A20 from cohort A, as well as readers B2 and B9 from cohort B, were linked to a higher incidence of false positives, it was noteworthy that they possessed substantial experience in mammography reading. All of these readers had accumulated years of experience that surpassed the median level within their respective cohorts. The remaining readers from each cohort shared more diagnostic agreement. For false negative errors, no outlying readers were found and all shared similarities in detecting malignancies for both cohorts. Based on HC analysis and dendrograms, we excluded readers A4, A10, A12, A14, A15, and A20 from cohort A and B2 and B9 from cohort B and their annotations from analysing false positive errors (ie, 14 radiologists in cohort A and 14 radiologists in cohort B, respectively), while these data were preserved for analysis of false negative errors (ie, 20 radiologists in cohort A and 16 radiologists in cohort B, respectively).

After omitting the readers with different reading patterns, we found a total of 207 (CC: 97; MLO: 110) and 241 (CC: 95; MLO: 146) normal areas that were reported as cancer for observers from cohorts A and B, respectively. Each area’s false positive response ranged from 1 to 10 for the remaining 14 readers from cohort A (CC: 1-8; MLO: 1-), and from 1 to 7 for the remaining 14 readers from cohort B (CC: 1-7; MLO: 1-7), with the median value at one for both cohorts. Based on the criteria mentioned in Section “Diagnostic error analysis”, normal areas with a false response greater than one were defined as “difficult” and those with one false response were classified as “easy”.

For the 42 cancer-containing areas (21 lesions × two projections), we found each area’s false negative response ranged from 3 to 20 for the 20 radiologists from cohort A (CC: 3-20; MLO: 4-20), and from 1 to 16 for the 16 readers from cohort B (CC: 2-16; MLO: 1-16), with the median values at 12 and 7.5 for cohort A and cohort B, respectively. According to the criteria mentioned in Section “Diagnostic error analysis”, the cancer-containing areas missed by more than 12 readers for cohort A and those of more than seven readers for cohort B were classified as “difficult”, and the remaining areas were classified as “easy”.

Table 2 shows the performance of cohort-based models predicting challenging mammographic areas which are most likely to be associated with an error for readers of cohort A and cohort B. It shows that all models predicting the false positive errors performed significantly better than random guessing with AUC values greater than 0.5, meaning that the cohort-based model can be used to analyse and predict the common false positives for specific reader cohorts based on the image-derived radiomic features. However, the performance of models predicting false negative errors was inconsistent with a wider range of possible AUC values. This is highly likely due to the small size used to train the RF model.

**Table 2.** Model performance in detecting difficult local areas using image-derived radiomic features for observers from cohorts A and B.

Error types	Cohort	View	AUC (95% CI)	Accuracy
False positive errors	Cohort A	CC	0.864 (0.803-0.918)	0.815
		MLO	0.829 (0.749-0.893)	0.801
	Cohort B	CC	0.652 (0.590-0.756)	0.729
		MLO	0.747 (0.620-0.861)	0.849
False negative errors	Cohort A	CC	0.677 (0.418-0.923)	0.714
		MLO	0.673 (0.407-0.907)	0.524
	Cohort B	CC	0.600 (0.300-0.900)	0.700
		MLO	0.505 (0.235-0.773)	0.476

a CC means craniocaudal and MLO means mediolateral oblique projection.

The results presented in Table 3 demonstrated that excluding outlying readers did not significantly impact the model performance, as indicated by the *P*-values for both cohorts and views. The AUC values for models excluding outliers were generally comparable to those including all readers.

**Table 3.** Exploring the impact of removing outlying readers on model performance.

Cohort	View	Without outlying readers	With all readers	*Z* score	*P*-value
Cohort A	CC	0.863 (0.802-0.918)	0.807 (0.694-0.909)	0.899	.369
	MLO	0.829 (0.749-0.893)	0.799 (0.699-0.883)	0.503	.615
Cohort B	CC	0.652 (0.498-0.756)	0.609 (0.393-0.802)	0.349	.727
	MLO	0.747 (0.620-0.861)	0.781 (0.626-0.912)	−0.356	.722

**Figure 4.**
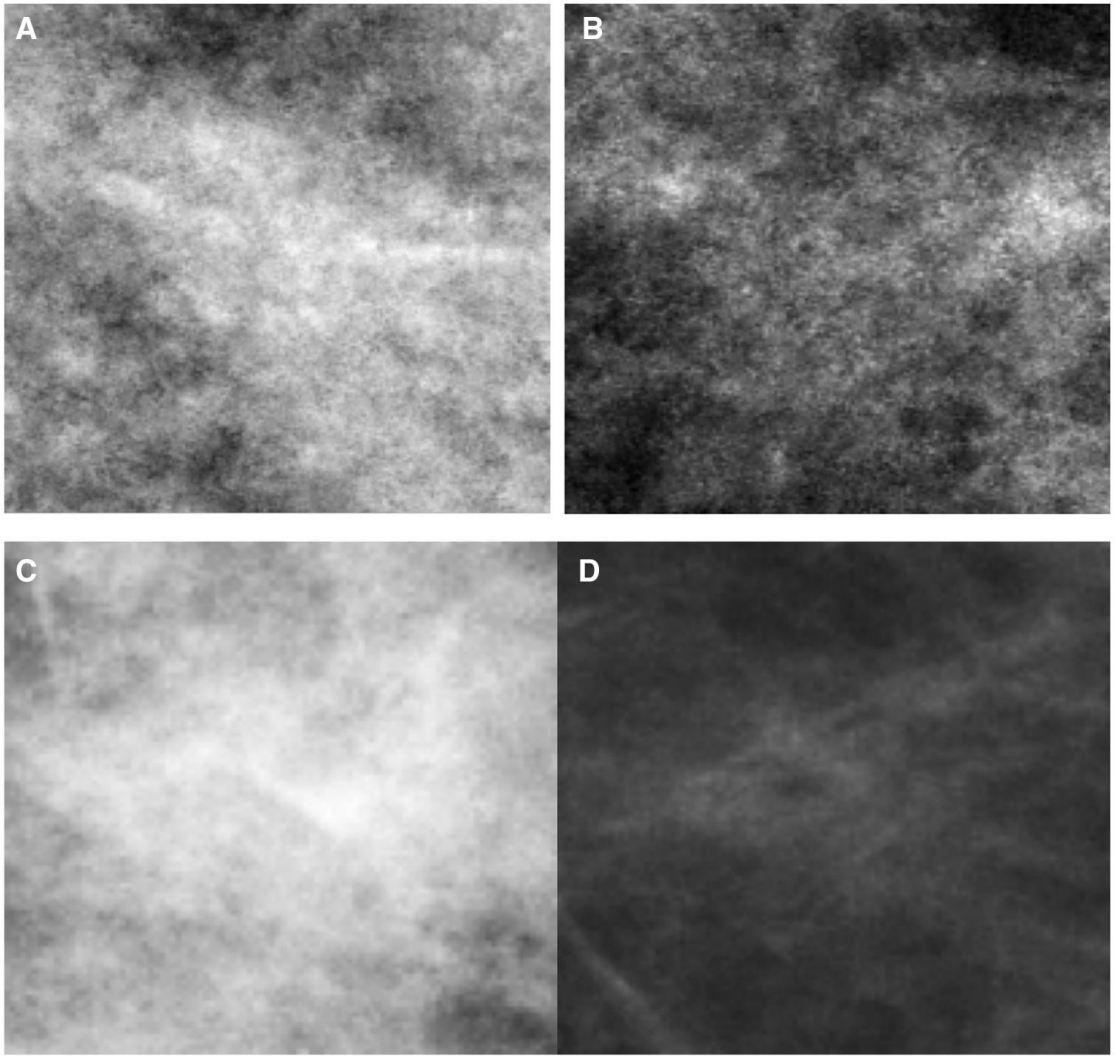
Illustration of mammographic appearance being correctly and incorrectly classified by our proposed model. (A) The “difficult” to interpret cancer-free patch for cohort A being correctly classified (true positive); (B) the “difficult” to interpret cancer-free patch for cohort A being incorrectly classified (false negative); (C) the “difficult” to interpret cancer patch for cohort B being correctly classified (true positive); and (D) the “difficult” to interpret cancer patch for cohort B being incorrectly classified (false negative).

Figure 4 displays examples of mammographic appearances being correctly and incorrectly classified by our proposed model. Notably, our model exhibited a tendency to classify areas with heterogeneous intensity values as “difficult” to interpret, while areas with fewer intensity variations were categorized as “easy” to interpret. This finding aligns with AL Mousa et al’s eye-tracking research, where they observed that the perceptual difficulty of a lesion, evidenced by increased dwell time and more fixations, was mainly influenced by whether the lesions were situated within dense fibroglandular tissue.^58^

Table 4 presents the top five discriminative radiomic features that contributed significantly to each classification pipeline. We can see that the Gobar- and MR-filter-based statistics and features from GLCM were frequently selected as important attributes. The discriminatory power of these features was evaluated *via* the Mann-Whitney *U* test and we found some features’ distribution was statistically different between the two difficulty categories (Table 4). Specifically, for the models analysing false positive errors, the mammographic regions with higher values in the Gabor and MR outputs are more challenging to interpret correctly for readers in both cohorts; in cohort A, regions with higher “*grey level nonuniformity*” were also particularly difficult, indicating that the areas displaying heterogenous intensity values are more frequently linked to false positives than those with homogeneous grey levels.

**Table 4.** Comparison of discriminative features between “easy” and “difficult” mammographic ROIs using the Mann-Whitney *U* test.

Error types	Cohorts	Discriminative features	Easy group	Difficult group	*P*-values
False positive errors	Cohort A	Gabor-filtered output at wavelength equals to 4, orientation equals to 0°	3265.802 (2100.658-4666.417)	4793.386 (2616.979-6320.890)	.031*
		MR-filter based: maximum bar-filter response across all orientations (0°, 30°, 60°, 90°, 120°, and 150°) at scale of (1, 3)	118.017 (76.791-167.764)	171.325 (102.979-208.339)	.026*
		Gabor-filtered output at wavelength equals to 4, orientation equals to 90°	3510.685 (2216.429-4635.308)	4501.406 (2919.227-5908.272)	.069
		GLRLM: grey level nonuniformity	2722.468 (1794.484-3628.297)	3861.969 (2552.938-5499.328)	.008*
		Gabor-filtered output at wavelength equals to 4, orientation equals to 60°	3304.671 (2071.810-4584.962)	4498.769 (2876.927-5577.565)	.031*
	Cohort B	Gabor-filter based: Gabor-filtered output at wavelength equals to 4 and orientation equals to 0°	2535.157 (1743.628-2947.566)	3712.736 (2357.581-4214.231)	.005*
		MR-filter based: maximum bar-filter response across all orientations (0°, 30°, 60°, 90°, 120°, and 150°) at scale of (2, 6)	90.296 (63.230-116.064)	128.523 (92.185-153.279)	.005*
		Gabor-filter based: Gabor-filtered output at wavelength equals to 4 and orientation equals to 150°	2461.576 (1630.121-2861.701)	3443.517 (2242.214-3959.307)	.004**
		GLCM: sum average (offset = 3)	6.869 (6.594-7.067)	6.988 (6.863-7.106)	.407
		First-order: the minimum intensity value	3.844 (0.000-8.611)	2.391 (0.086-3.672)	.990
False negative errors	Cohort A	GLCM: information measure of correlation 2 (offset = 1)	99293.655 (47020.846-158332.192)	29180.237 (6106.387-39617.539)	.034*
		MR-filter based: response from Laplacian of Gaussian-filter oriented at six orientations (0°, 30°, 60°, 90°, 120°, and 150°) at each scale [(1, 3), (2, 6), (4, 12)]	−76.787 (−89.691 to −30.323)	−31.034 (−39.753 to 4.275)	.189
		GLCM: energy (offset = 1)	39.584 (29.286-46.205)	21.967 (18.363-26.123)	.014*
		GLCM: difference variance (offset = 1)	146779.633 (70722.524-257921.784)	45735.465 (160333.678-64691.755)	.027*
		GLCM: cluster shade (offset = 9)	2.592 (2.451-2.672)	2.315 (2.211-2.405)	.014*
	Cohort B	GLDS: energy (3 × 3 neighbourhood)	0.038 (0.037-0.040)	0.047 (0.040-0.060)	.159
		Law’s: average texture energy from LS and SL kernels (5 × 5 neighbourhood)	62.056 (57.831-64.170)	50.817 (42.406-63.935)	.566
		Law’s: average texture energy from LS and SL kernels (5 × 5 neighbourhood)	198.204 (193.201-211.351)	167.165 (132.404-213.208)	.310
		GLCM: sum entropy (offset = 5)	0.376 (0.366-0.385)	0.415 (0.382-0.471)	.159
		GLDS: mean (5 × 5 neighbourhood)	14.087 (13.483-15.637)	11.315 (9.832-13.161)	.111

Data are given as median and interquartile range.

*P*-values are displayed after Bonferroni correction.

* *P *<* *.05.

** *P *<* *.005.

For models investigating false negative errors, the “*information measure of correlation 2*”, “*energy*”, “*difference variance*”, and “*cluster shade*” from GLCM were statistically higher in the “easy” class for readers from cohort A, indicating that it is easier to detect cancers with a noticeable intensity variance in relatively uniform grey level surroundings. However, for radiologists in cohort B, none of the features selected by the pipeline showed statistically significant differences between the two classes. Despite this, the slightly higher “*energy*” (from GLDS) and “*sum entropy*” (from GLCM) and lower “*Law's measures*” and “*mean*” (from GLDS) in the “difficult” group suggested that cancers with indefinite edges and spots in a relatively coarse background with great intensity changes were more likely to be missed by readers from cohort B.

## Discussion

In this study, we retrospectively analysed the reading data of radiologists from two different geographic populations that evaluated an identical test set containing dense screening mammograms and proposed machine learning pipelines for predicting diagnostic errors using image-derived radiomic characteristics. Our findings revealed that radiologists with similar geographic characteristics, regardless of the variation in reading expertise, share comparable error-making patterns when evaluating dense mammograms, and these regularities can be successfully predicted by our presented pipelines. This finding has profound implications for future research on cohort-customized radiology training, guiding the development of tailored education programs in mammography interpretation. Such programs might incorporate challenging mammography cases tailored to specific reader cohorts based on their shared error patterns captured by our proposed pipeline.

To explore whether the reading behaviours of radiologists with matching geographic characteristics are comparable, we first analysed the readers’ responses to all suspicious local regions, including the normal areas suspected of cancer and the malignant areas using agglomerative HC. By doing this, the readers were iteratively clustered based on diagnostic agreement, and those with the most dissimilar interpretations formed a branch on its own. We found some readers within the same population can be clustered into the same branch, suggesting a comparable interpretation patterns when detecting malignancies and identifying normal appearances. While acknowledging that other confounding factors may have influenced our results, this finding underscores the importance of considering geographical factors in mammography interpretation and highlights the need for region-specific training to address these variations and improve diagnostic accuracy. The difficulty in interpreting dense mammograms is further confirmed by our findings. In dense mammography, cancers are masked by superimposed normal tissues, leading to a higher probability of false negatives, so it is more likely for the readers to, in effect, “reach agreement” to miss the lesion.^59^ On the other hand, the superposition of the radiopaque normal tissues creates new characteristics that can mimic malignancies, causing several suspicious regions on the mammogram.^59^ As missing malignancies will lead to delayed treatment and poor health outcomes, some readers may be more conservative to not miss any suspicious appearances. However, it is worth noting that the higher false positives might also be due to the laboratory effect, meaning that the reading behaviour in evaluating a test set may not reflect performance in actual clinical practice. That is also why we excluded these outlying readers from model construction.

The rationale for employing HC and excluding outlier readers was to improve the generalizability of the proposed cohort-based model. This involved identifying radiologists whose reading patterns diverged significantly from their cohort peers and subsequently excluding them from model development. However, comparing the performance of models of all included readers versus the removed outliers revealed no statistically significant differences. This suggests that the presence of outlier readers does not affect the accuracy of the models. Consequently, the models demonstrate reliable generalizability across a broader population of radiologists. It is worthwhile to note, however, our study utilized internal validation, which may contribute to the lack of observed differences. Future studies should incorporate external validation to further assess the model’s performance across diverse datasets and settings. This external validation could provide additional confidence in the model’s applicability beyond the specific cohorts and dataset used in our study.

To further investigate whether particular mammographic appearances are more likely to induce diagnostic errors for a specific radiologist cohort, we built classification models to compare the radiomic features extracted from local areas with low and high perceived difficulty levels. We found that the models can successfully distinguish the normal areas that are likely to be reported as cancer from those that most readers from a specific cohort can diagnose correctly. Identifying normalities accurately is important in reading screening mammograms as most cases will be normal in the screening setting. Additionally, by analysing the discriminative features, we found that the normal regions with higher Gabor and MR responses were more likely to be reported as cancer on dense mammograms for readers from both populations, despite a cross-cohort variation in the directions and scales where the maximum responses occurred. It is not surprising that Gabor filters were evaluated with great discriminatory power in this classification task, as these filters were suggested to approximate mammals’ visual receptive field profiles and are widely used in edge detection and texture analysis.^60^ The Gabor and MR filters are resistant to changes in object rotation and scale by convolving the image with an comprehensive list of filters with different directions and scales, and filter with the highest output has an orientation and scale similar to that of the investigated structures.^60^^,^^61^ Intuitively, greater energy values for the output of these filter banks show higher intensity variation along the specific direction^62^ and hence a distinct difference between the focal point of the false positive areas and its surroundings. Such structure would resemble an actual lesion and therefore, it would make it difficult for a reader to rule out this false positive area.

For the malignant areas, despite the inconsistent model performance likely due to the limited sample size, we found that tumours with homogenous surroundings were much easier to detect compared to those on a coarse background with significant intensity variations for both cohorts. However, the univariate analysis showed that the features considered important by the RF model did not show any statistical difference between the two difficulty categories in cohort B. This may be attributable to the Bonferroni correction applied, which is a conservative test to control false positive findings, or it could be explained that it is the interrelationship between features that contribute the most to the prediction. For cohort A, we observed that “cluster shade” exhibited higher values in the easy class. This indicates the presence of asymmetry in the distribution of grey level values within small neighbourhoods of pixels in the image, highlighting the tendency of pixel intensity values to cluster closely together. When “cluster shade” has a higher value, it suggests an increased concentration of pixel intensities around the mean or central value of the local region, leading to a more pronounced peak in the distribution. In contrast, a lower value implies a more spread-out and skewed distribution with pixel intensities less concentrated around the mean, resulting in a longer tail on one side. Intuitively, in the cases where “cluster shade” has a higher value (ie, more pronounced clustering), the lesions would be easier to detect as they exhibit a distinctive and localized concentration of pixel intensities. Such localized clustering makes the lesions visually stand out from their surroundings, aiding radiologists in identifying these abnormalities more effectively during mammogram analysis. On the other hand, lower “cluster shade” values may indicate a more scattered distribution of intensities, making it potentially harder for radiologists to discern and accurately identify lesions in the mammograms due to the lack of distinctive concentration patterns. In cohort A, easy lesions also exhibited higher “*information measure of correlation 2*”. This feature specifically focuses on the correlation between pairs of pixel intensities and measures the similarity of grey level values for pixel pairs along the specified direction. It is called an “Information Measure” because it quantifies the amount of information shared or correlated between the two pixels under consideration. It provides insights into how much one pixel’s intensity value can be predicted or inferred based on the intensity value of the other pixel within the defined spatial context. Its higher value indicates a stronger linear correlation between the pixel intensities of the paired pixels; hence, the pixel intensities are more strongly related or co-varying along that specific direction. When it has a lower value, it implies that intensity values of the two pixels are less dependent on each other, showing less predictable patterns along the specified direction. When radiologists review mammograms, they rely on identifying recognizable patterns and distinctive features to detect abnormalities. Easier lesions with higher “*information measure of correlation 2*” values likely exhibit more predictable and coherent texture patterns, making them visually distinct and easier to identify during the analysis. In contrast, more challenging lesions with lower “*information measure of correlation 2*” values may exhibit irregular and less organized texture patterns. These regions might contain heterogeneous structures or overlapping tissue, leading to weaker linear correlation between pixel intensities. Consequently, these areas could appear more visually cluttered and harder for radiologists to distinguish potential abnormalities from the surrounding breast tissue. For this cohort, easier lesions were also likely to have higher energy values. These easier lesions typically exhibit higher contrast compared to the surrounding breast tissue. The presence of pronounced intensity variations within the lesion enhances the local homogeneity, leading to an increase in the number of repeated intensity pairs in the GLCM and, in turn, resulting in higher energy. In cohort B, none of the features had a significant impact on lesion detectability. This lack of significance might be attributed to their extensive experience, which allowed them to develop a well-established understanding of malignant lesion appearances. As a result, their perceptual process became more resilient and less influenced by variations in image features. These findings emphasized the clinical relevance of certain radiomic features in mammographic interpretation. Features like “cluster shade”, “information measure of correlation 2”, and energy provide insights into the textural characteristics of lesions that aid radiologists in detecting abnormalities. Understanding these relationships can improve diagnostic accuracy and potentially lead to better clinical decision-making in breast cancer detection.

As we proved that the interpretation behaviours of radiologists with comparable geographic characteristics were not random but formed a pattern, and such regularities can be represented by our proposed models, it is feasible to develop cohort-customized education programs in mammography interpretation by providing more challenging mammography cases for specific reader cohorts. Traditionally, mammography training takes place during residency years when radiologist trainees are mentored by senior members. However, significant drawbacks associated with this type of training include the limited number of malignant cases encountered and the delayed feedback on performance, often not provided until the annual audit if they are screen reading.^63^^,^^64^ To address these limitations, online mammography training programs have gradually gained popularity. For example, the BREAST is an online mammography training platform that provides self-assessment test sets containing a great number of mammograms with a higher prevalence of cancer cases than the screening population, and readers can receive instant individual feedback once they completed assessing all the mammograms in a test set.^20^ However, most available online programs are still constructed as a one-size-fits-all without necessarily considering the readers’ individual and group error patterns. Ideally, mammography test sets should be customized according to the readers’ specific weaknesses. It is feasible for our radiomic pipeline to be incorporated into the selection of training materials for the development of cohort-specific test sets. We can apply the proposed pipeline to a mammography database, and the model will automatically search each case for any difficult signs. Then, the cases enriched with challenging appearances can be collected to form a tailored test set for a specific reader cohort.

This preliminary study has limitations. Firstly, all the mammograms in our test set originated from the Australian population, albeit with an oversampling of high dense appearances to simulate the breast density encountered by Chinese radiologists.^65^ Consequently, it was undeniable that the Australian cohort may possess a higher degree of familiarity with the test set compared to their Chinese counterparts. This familiarity could potentially contribute to lower variability among the readers within cohort and result in better diagnostic performance. To validate the effectiveness of our proposed approach, we strongly advocate for future studies to be conducted on mammograms that accurately represent the populations encountered by the readers in their clinical practice. This could entail collecting reader performance data from Chinese radiologists assessing mammograms specifically from the Chinese population.

Additionally, the findings of this study should be used with caution when applied to radiologists from different populations with varying mammography interpretation behaviours, to mammograms acquired using various imaging technologies, and to different clinical environments. Future studies should validate the proposed mythology with a more diverse range of mammography readers. Differences in training, cultural practices, and clinical guidelines can lead to variations in how radiologists interpret mammograms. Therefore, it is crucial to test the model with radiologists from different demographic and professional backgrounds to ensure its generalizability in a wider range of clinical environments. Furthermore, imaging technology, such as the mammography manufacturer and image acquisition techniques, may impact the model's generalizability. Variations in imaging technology or acquisition protocols (eg, resolution, contrast, and noise levels) can alter the radiomic features extracted from mammograms. Although we performed image preprocessing to standardize variations across different acquisition processes, future research should further test the model across various imaging platforms to ensure its robustness. The clinical environment, including the expertise of the radiologists, the diagnostic workflow, and the type of healthcare facility (eg, community hospitals versus specialized cancer centres), can also influence the interpretation of mammograms and the application of our proposed radiomic models. These variations in clinical environment and professional expertise underscore the importance of validating our model in diverse clinical settings. We expect future studies to test and refine the model across various healthcare facilities and radiologist expertise levels.

Another limitation pertains to the methodology used for clustering diagnostic annotations. While we implemented a blinding protocol and second reader validation, some degree of subjectivity may persist in manual adjustments due to the interpretive nature of this task. Furthermore, as a preliminary study, we only investigated the effectiveness of the RF built-in feature selection method in predicting diagnostic errors. Alternative methods such as mutual information, recursive feature elimination (RFE), or LASSO regularization could potentially yield different sets of discriminative features. We acknowledge that the choice of method can impact model interpretability and generalization performance and hope that future studies will continue to explore these methodologies to enhance their application in diverse contexts. Finally, the reading behaviour of radiologists in reading a test set with a higher prevalence of cancer could differ from that in clinical practice due to the laboratory effect. This might also be the reason that why some radiologists contributed to a higher number of false positives than the rest from the same cohort in HC analysis. However, we excluded the readers with the most dissimilar reading patterns in this study to enhance the generality of the models. In addition, this problem can be addressed by tuning the difficulty threshold in actual clinical practices, and it is worthwhile for future studies to investigate the diagnostic behaviours of radiologists in the real world.

## Conclusions

The cohort-based AI pipeline can successfully map the local mammographic appearances to the cohort-specific diagnostic errors in reading dense screening mammograms and has the capability of predicting the common false positive errors for specific reader cohorts based on the image-derived radiomic features. The proposed method can be further validated and applied to develop more challenging mammographic cases for group-tailored training to improve future mammography interpretation accuracy.

## Supplementary Material

tqae195_Supplementary_Data

## Data Availability

The data presented in this study are available on request from the corresponding author. The data are not publicly available due to institutional regulations.
