# Supplementary material for: Radiomic analysis of cohort-specific diagnostic errors in reading dense mammograms using artificial intelligence
Source: Br J Radiol. 2024 Oct 9;98(1165):75–88. doi: 10.1093/bjr/tqae195 (PMC11652727; doi:10.1093/bjr/tqae195)
Supplement: tqae195_Supplementary_Data [file tqae195_supplementary_data.docx]

TITLE: Radiomic Analysis of Cohort-Specific Diagnostic Errors in Reading Dense Mammograms Using Artificial Intelligence

**Supplementary table 1.** Radiomic features used in this study.

| Feature type | | Feature name |
| --- | --- | --- |
| First-order (28) |  | Mean,standard deviation, skewness, kurtosis,minimum, maximum,  percentile (5th , 10th , 15th , 20th , 25th , 50th , 75th , 80th , 85th , 90th , and 95th) ,  intensity percentile range (100-0, 99-1, and 95-5),  mean*, variance*, median*, mode*,skewness*, kurtosis*, energy*, entropy* |
| Textural (139) | Gray level co-occurence matrix (GLCM) | Autocorrelation, contrast, correlation1, correlation2, cluster prominence, cluster shade, dissimilarity, enegy, entropy, homogeneity1, homogeneity2, maximum likelihood, sum of squares of variance, sum average, sum variance, sum entropy, difference variance, information measures of correlation1, information measures of correlation2, inverse difference normalized, inverse difference moment normalized   orientation = 0, 45, 90, 135 (averaging along four directions) pixel distance = 1, 3, 5, 9 number of levels = 64 |
|  | Gray level run length matrix  (GLRLM) | Short runs emphasis, long runs emphasis, grey level nonuniformity, run percentage, run length nonuniformity, low grey-level run emphasis, high gray-level run emphasis  Offset = 1, 2, 3, 4 |
|  | Gray level difference statistics  (GLDS) | Homogeneity, contrast, energy, entropy, mean  Direction = 0, 45, 90, 135 |
|  | Neighborhood gray tone difference matrix (NGTDM) | Coarseness, contrast, busyness, complexity, strength  Neighborhood size = 3, 5, 9 |
|  | Statistical feature matrix (SFM) | Coarseness measure, contrast measure, periodicity measure, roughness measure  Neighborhood size = 4, 8 |
|  | Gray-level sharpness measure  (GLSM) | Gradient, laplacian, wavelet decomposition (level 2 and 3 wavelet decomposition, db6 wavelet) |
|  | Fractional dimension texture analysis | Roughness |
| Transform-based (34) | Laws texture energy measures | Texture energy from LL kernel, texture energy from EE kernel, texture energy from SS kernel, average texture energy from LE and EL kernels, average texture energy from ES and SE kernels, average texture energy from LS ans SL kernels  Filter size = 3, 5, 7 |
|  | Gabor texture features | Wavelengh = 4 Orientation = 0, 30, 60, 90, 120, 150 |
|  | MR filter-based features | Filter size = 49 Scale = 1, 2, 4 Orientation = 0, 30, 60, 90, 120, 150 |
|  | Fourier transform spectrum | Radial sum, angular sum |
